# *LncRSPH9-4* Facilitates Meningitic *Escherichia coli*-Caused Blood–Brain Barrier Disruption via *miR-17-5p*/MMP3 Axis

**DOI:** 10.3390/ijms22126343

**Published:** 2021-06-14

**Authors:** Bojie Xu, Ruicheng Yang, Jiyang Fu, Bo Yang, Jiaqi Chen, Chen Tan, Huanchun Chen, Xiangru Wang

**Affiliations:** 1 State Key Laboratory of Agricultural Microbiology, College of Veterinary Medicine, Huazhong Agricultural University, Wuhan 430070, China; pochieh@webmail.hzau.edu.cn (B.X.); yangruicheng@mail.hzau.edu.cn (R.Y.); fujy@webmail.hzau.edu.cn (J.F.); ybtc@webmail.hzau.edu.cn (B.Y.); chenjiaqi@webmail.hzau.edu.cn (J.C.); tanchen@mail.hzau.edu.cn (C.T.); chenhch@mail.hzau.edu.cn (H.C.); 2 Key Laboratory of Preventive Veterinary Medicine in Hubei Province, The Cooperative Innovation Center for Sustainable Pig Production, Wuhan 430070, China; 3 Key Laboratory of Development of Veterinary Diagnostic Products, Ministry of Agriculture of the People’s Republic of China, Wuhan 430070, China; 4 International Research Center for Animal Disease, Ministry of Science and Technology of the People’s Republic of China, Wuhan 430070, China

**Keywords:** brain microvascular endothelial cells, *lncRSPH9-4*, *miR-17-5p*, MMP3, tight junctions

## Abstract

Brain microvascular endothelial cells (BMECs) constitute the structural and functional basis for the blood–brain barrier (BBB) and play essential roles in bacterial meningitis. Although the BBB integrity regulation has been under extensive investigation, there is little knowledge regarding the roles of long non-coding RNAs (lncRNAs) in this event. The present study aimed to investigate the roles of one potential lncRNA, *lncRSPH9-4*, in meningitic *E. coli* infection of BMECs. *LncRSPH9-4* was cytoplasm located and significantly up-regulated in meningitic *E. coli*-infected hBMECs. Electrical cell-substrate impedance sensing (ECIS) measurement and Western blot assay demonstrated *lncRSPH9-4* overexpression in hBMECs mediated the BBB integrity disruption. By RNA-sequencing analysis, 639 mRNAs and 299 miRNAs were significantly differentiated in response to lncRSPH9-4 overexpression. We further found *lncRSPH9-4* regulated the permeability in hBMECs by competitively sponging *miR-17-5p*, thereby increasing MMP3 expression, which targeted the intercellular tight junctions. Here we reported the infection-induced *lncRSPH9-4* aggravated disruption of the tight junctions in hBMECs, probably through the *miR-17-5p*/MMP3 axis. This finding provides new insights into the function of lncRNAs in BBB integrity during meningitic *E. coli* infection and provides the novel nucleic acid targets for future treatment of bacterial meningitis.

## 1. Introduction

Bacterial meningitis is the most important life-threatening infection of the central nervous system (CNS) with high morbidity and mortality and *Escherichia coli* is the most common gram-negative pathogenic bacterium causing this outcome [1]. Most bacterial meningitis develops from bacterial penetration of the BBB [2]. Vascular endothelium constitutes the structural and functional basis of the BBB and plays an important role in maintaining the integrity of the BBB, as well as CNS homeostasis [3]. BMECs are characterized by the presence of tight junction proteins (TJs), including Claudins, Occludin and zonula-occludens [4,5]. BMECs dysfunction is often caused by the decrease or re-distribution of these TJs, which lead to disruption of the BBB [6]. Thus, protecting and maintaining the BBB function is of positive significance in alleviating brain damage after the CNS-invading pathogens infection.

LncRNAs are considered regulators of diverse biological processes, including imprinting control, cell differentiation and development [7,8,9], as well as many pathological processes, such as cancer, chronic inflammation and infectious diseases [10,11,12]. Aberrant expression and mutations of lncRNAs uncovered in various brain dysfunctions have led researchers to investigate the potential roles of lncRNAs in brain physiology and pathology [13]. For example, lncRNAs *MALAT1 GAS5* and *NEAT1* are widely recognized to be implicated in cancer, vascular diseases and neurological disorders [14,15,16,17]. *MALAT1* was reported to promote the BMEC autophagy; *NEAT1* was able to activate the NF-κB signaling pathway in nerve cells [17,18]. Regarding lncRNAs, the competing endogenous RNA (ceRNA) working mechanism has been largely evidenced, in which lncRNAs act as the ceRNA to interact competitively with miRNAs and regulate the expression of the target proteins [19]. Our previous lncRNA transcriptomics sequencing identified a batch of significantly different lncRNAs in hBMECs upon meningitic *E. coli* infection [20] and we further noticed one novel lncRNA, *lncRSPH9-4*, that was significantly up-regulated along with the infection. We focused on this lncRNA because we found that overexpressing *lncRSPH9-4* in hBMECs would cause a decrease of the impedance values. However, the specific working mechanism of this lncRNA in the pathogenic process is unclear.

A variety of miRNAs have been reported to regulate the integrity of the BBB, such as *miR-18a*, *miR-338-3p* and *miR-182* [13,21,22]. In the current study, we also focused on a miRNA, *miR-17-5p*, which is a member of the *miRNA-17-92* cluster and has been reported in several cancers and CNS dysfunctions, such as ischemia-reperfusion injury and neurodegenerative disease [23,24]. More importantly, we found that *lncRSPH9-4* can specifically sponge *miR-17-5p* by ceRNA analysis. Whether this miRNA was rightly involved in *lncRSPH9-4* regulation of the hBMECs barrier function will be elucidated in this work.

## 2. Results

### 2.1. LncRSPH9-4 Was Significantly Up-Regulated and Cytoplasmically Distributed in Meningitic E. coli-Challenged hBMECs

**Figure 1:**
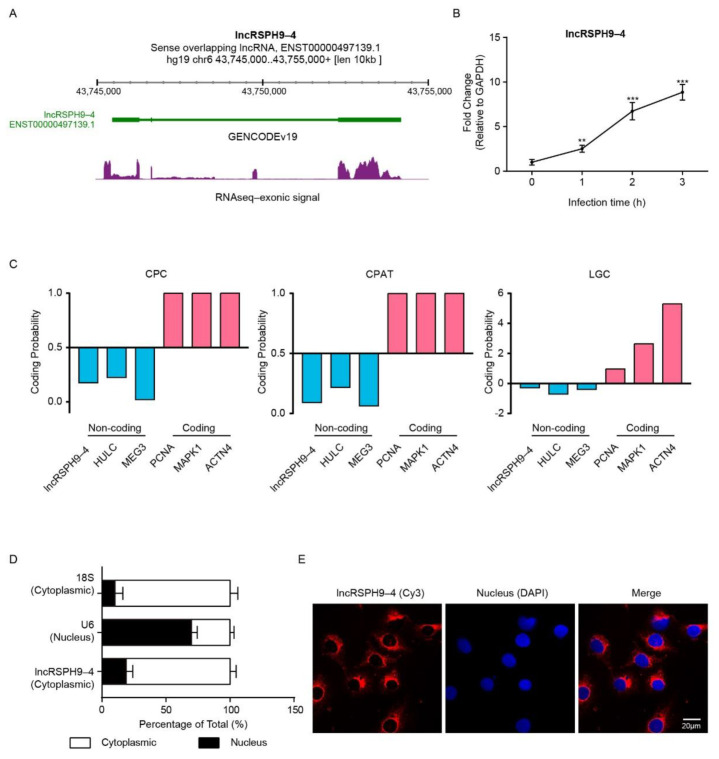
The expression and subcellular localization of *lncRSPH9-4*. (**A**) Visualization of *lncRSPH9-4* in ZENBU browser, which showed exonic expression signal in several different visualizations. (**B**) *LncRSPH9-4* expression in hBMECs was measured using qPCR after meningitic *E. coli* infection for 0 h, 1 h, 2 h and 3 h. *GAPDH* was used as the reference control. ** indicated *p* < 0.01 and *** indicated *p* < 0.001 by one-way ANOVA analysis. (**C**) The prediction of *lncRSPH9-4* coding potential by using CPC, CPAT and LGC. (**D**) Nuclear/Cytoplasmic localization analyses of *lncRSPH9-4* in hBMECs by qPCR. The *18S* and *U6* distribution were selected as the cytoplasmic control and the nuclear control, respectively. (**E**) Subcellular localization of *lncRSPH9-4* in hBMECs by FISH assay. Red, Cy3-labeled positive hybridization signals; Blue, DAPI-stained nucleus. Scale bars, 20 μm.

Based on our previous study, a total of 895 lncRNAs in hBMECs exhibited significant differences in response to meningitic *E. coli* infection [20], among which one lncRNA, *lncRSPH9-4*, was included and focused on in the current study. *LncRSPH9-4*, a sense overlapping lncRNA, was located on chromosome 6 of the genome (Figure 1A). It was significantly and time-dependently up-regulated in hBMECs, when challenged with meningitic *E. coli* (Figure 1B).

Next, three tools, including Coding Potential Caculator (CPC), Coding-Potential Assessment Tool (CAPT), ORF Length and GC content (LGC), were used to predict the protein-coding potential of *lncRSPH9-4*. The prediction results supported that *lncRSPH9-4* has no protein-coding potential, similar to the potential coding analyses of the known lncRNAs *HULC* and *MEG3*, but opposite to the typical protein-coding mRNAs like *PCNA*, *MAPK1* and *ACTN4* (Figure 1C). The subcellular localization of this lncRNA in hBMECs was next determined by both nucleocytoplasmic separation assay and fluorescence in situ hybridization (FISH). As shown in Figure 1D, *lncRSPH9-4* could be detected in both nuclear and cytoplasmic compartments but was highly enriched in the cytoplasm, which was similar to that of the 18S rRNA (Figure 1D). FISH results also supported the cytoplasmic distribution of this *lncRSPH9-4* in hBMECs (Figure 1E). Together, these data show that *lncRSPH9-4* is a cytoplasm-located lncRNA in hBMECs and is significantly up-regulated in response to meningitic *E. coli* infection.

### 2.2. LncRSPH9-4 Negatively Affected the Barrier Permeability of hBMECs

**Figure 2:**
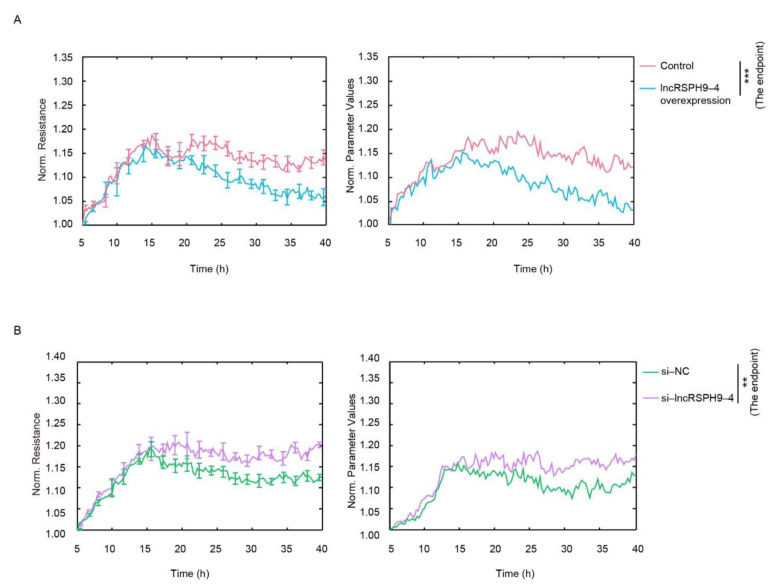
The impedance analyses of the hBMECs monolayer with *lncRSPH9-4* overexpression or knocking-down. (**A**) The impedance changes of hBMECs by the overexpression of *lncRSPH9-4*, monitored by the ECIS system. Data were collected and presented as mean ± SD from three replicated wells at each time point. *** *p* < 0.001. (**B**) The impedance changes of hBMECs with knocking-down of *lncRSPH9-4* determined by ECIS. Data were collected and presented as mean ± SD from three replicates at each time point. ** *p* < 0.01.

To study the function of *lncRSPH9-4* in the infected hBMECs, we first evaluated the possible influence of *lncRSPH9-4* on growth characters of monolayer hBMECs. By application of an ECIS system that tests impedance values, an important indicator of the monolayer permeability, we observed that overexpression of *lncRSPH9-4* significantly reduced the impedance values of the hBMECs monolayer after 20 h of growth (Figure 2A). In contrast, knocking-down of *lncRSPH9-4* by siRNA significantly enhanced the resistance of hBMECs (Figure 2B). These data suggested that *lncRSPH9-4* negatively affected the barrier resistance of the hBMECs monolayer.

### 2.3. Differential mRNAs Transcription in hBMECs with LncRSPH9-4 Overexpression

We next seek to explore the possible mechanism of *lncRSPH9-4* regulating cell barrier resistance. *LncRSPH9-4* was inserted into pcDNA3.1(+) to obtain the overexpression constructs (Figure S1A) and the overexpression efficiency was further verified by qPCR (Figure S1B). The total RNAs in hBMECs with *lnsRSPH9-4* overexpression were extracted and subjected to mRNA and miRNA sequencing with three replicates.

**Figure 3:**
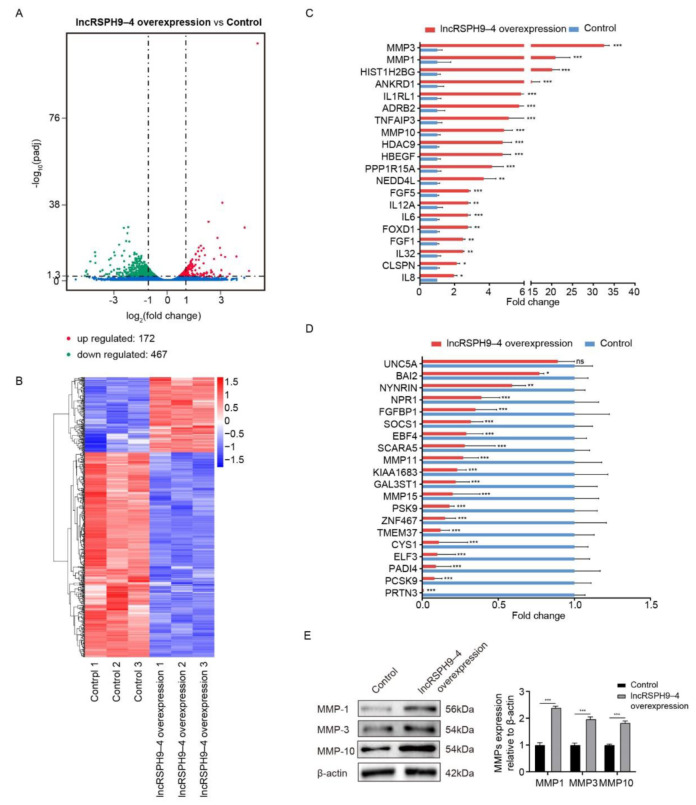
The mRNA transcription profiles in *lncRSPH9-4* overexpressed hBMECs. (**A**) Volcano plot showing the differentially expressed mRNAs in *lncRSPH9-4* overexpressed hBMECs compared with control cells. The red and green plots represent the significantly up-regulated and down-regulated mRNAs, respectively. (**B**) The heatmap showing the unsupervised clustering of significantly expressed mRNAs in hBMECs with or without *lncRSPH9-4* overexpression. (**C**,**D**) qPCR validation of the up-regulated (**C**) or down-regulated (**D**) mRNAs in hBMECs in response to *lncRSPH9-4* overexpression. *GAPDH* was used as the reference control. * *p* < 0.05, ** *p* < 0.01 and *** *p* < 0.001 by one-way ANOVA analysis. (**E**) Western blotting showed the significant up-regulation of the MMPs family members MMP1, MMP3 and MMP10. The β-actin was used as the loading control and densitometry was performed to analyze differences.

As the volcano map (Figure 3A) and heat map (Figure 3B) show, *lncRSPH9-4* overexpression resulted in a total of 639 mRNAs that significantly changed (increase by ≥2-fold, or decrease to ≤0.5-fold, at *p* ≤ 0.05), including 172 up-regulated mRNAs and 467 down-regulated mRNAs. Forty differentially altered mRNAs with the most significant changes were chosen to verify their expression levels by qPCR. The results demonstrated that these up-regulated or down-regulated mRNAs exhibited similar alteration trends as obtained from sequencing data (Figure 3C,D). Noticeably, as shown in Figure 3C, the MMPs family exhibited significant up-regulation after *lncRSPH9-4* overexpression. Therefore, the expression of MMP3, MMP1 and MMP10 were verified by Western blot analysis (Figure 3E). Meanwhile, the functional enrichment analysis revealed that the *lncRSPH9-4* overexpression-enriched differential mRNAs were involved in several canonical pathways, such as the TNF signaling pathway, PPAR signaling pathway, MAPK signaling pathway, focal adhesion and ECM-receptor interaction, etc., which have previously been reported to regulate the barrier function of endothelial cells (Figure S2).

### 2.4. Altered miRNAs Profiles in hBMECs with LncRSPH9-4 Overexpression

**Figure 4:**
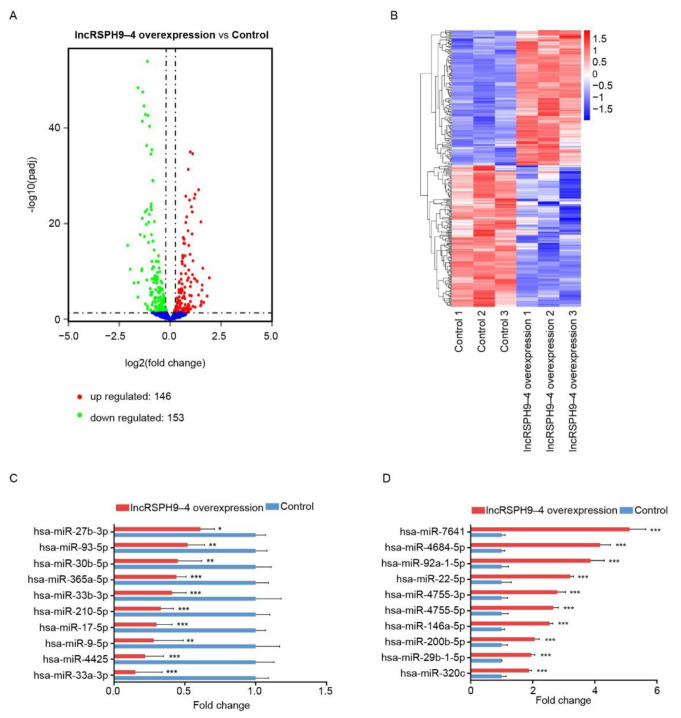
The miRNA transcription profiles in *lncRSPH9-4* overexpressed hBMECs. (**A**) Volcano plot exhibiting the differentially expressed miRNAs in hBMECs in response to *lncRSPH9-4* overexpression. The red and green plots represent the significantly up-regulated and down-regulated miRNAs, respectively. (**B**) Heatmap showing the unsupervised clustering of significantly changed miRNAs in hBMECs with or without *lncRSPH9-4* overexpression. (**C**,**D**) qPCR validation of the down-regulated or up-regulated miRNAs in hBMECs with the overexpression of lncRSPH9-4. *U6* was used as the reference control. * *p* < 0.05, ** *p* < 0.01 and *** *p* < 0.001.

The miRNAs profiles in response to *lncRSPH9-4* overexpression were also characterized. As shown, 153 miRNAs (decrease to <0.87-fold, at *p* ≤ 0.05) were significantly down-regulated and 146 miRNAs were significantly up-regulated (increase by >1.13-fold, at *p* ≤ 0.05) (Figure 4A,B). Among these, twenty miRNAs with the most significant changes were selected and verified by qPCR. They all showed the same alteration trends after *lncRSPH9-4* overexpression as that in miRNA sequencing data (Figure 4C,D). Similarly, functional enrichment analyses supported that they were widely involved in multiple regulative pathways and diverse processes, such as the VEGF signaling pathway, NOD-like receptor signaling pathway, MAPK signaling pathway, etc. (Figure S3). Noticeably, some pathways, such as the VEGF signaling and MAPK signaling ones have been previously evidenced to be associated with hBMECs permeability regulation. Combining both lncRNAs and miRNAs analyses, we thus presumed that *lncRSPH9-4* might work as the potential ceRNA to affect the barrier resistance of hBMECs.

### 2.5. The Competitive Endogenous RNA Network Analysis of LncRSPH9-4

**Figure 5:**
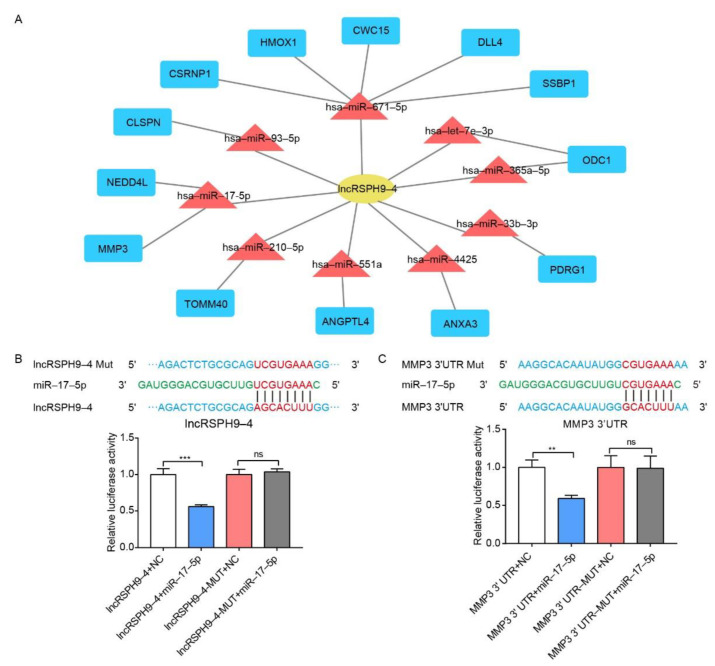
The competing endogenous RNA network of *lncRSPH9-4*. (**A**) Regulatory network analysis of *lncRSPH9-4* associated with significantly changed mRNAs and miRNAs based on ceRNA theory. (**B**) Predicted binding sites between *lncRSPH9-4* and *miR-17-5p*. Dual-luciferase reporter assays showing that *miR-17-5p* significantly reduced the luciferase activity of the wild-type *lncRSPH9-4* construct but not of the mutant construct. Data were presented as mean ± SD from three independent experiments. *** *p* < 0.001; ns, not significant. (**C**) Predicted binding sites between the *MMP3* 3′UTR region and *miR-17-5p*. Dual-luciferase reporter assays showing that *miR-17-5p* significantly reduced the luciferase activity of the wild-type *MMP3* 3′UTR construct but not of its mutant construct. Data were presented as mean ± SD from three independent experiments. ** *p* < 0.01; ns, not significant.

Based on the ceRNA theory, we subsequently constructed a ceRNA regulatory network that centered on *lncRSPH9-4*. As exhibited in Figure 5A, a total of 9 significantly differential miRNAs and 13 significantly differential mRNAs were involved in the *lncRSPH9-4*-centered regulative network. These 9 miRNAs, *miR-93-5p*, *miR-671-5p*, *let-7e-3p*, *miR-365a-5p*, *miR-33b-3p*, *miR-4425*, *miR-551a*, *miR-210-5p* and *miR-17-5p*, were therefore predicted as the sponging miRNAs and statistically significantly correlated with *lncRSPH9-4* expression. Notably, one miRNA, *has-miR-17-5p*, was predicted to negatively regulate the expression of MMP3, suggesting that *lncRSPH9-4* may exert certain effects via sponging miRNAs and subsequently influencing the function of MMP3.

The interrelationship between *miR-17-5p* and *lncRSPH9-4* and that between *miR-17-5p* and *MMP3* were examined next, by luciferase report assay. As shown in Figure 5B, both the wild-type and mutant *lncRSPH9-4* (*LncRSPH9-4* MUT) were cloned into luciferase reporter plasmid psiCHECK2 and then co-transfected with or without *miR-17-5p* mimics. Likewise, both the wild-type *MMP3* and its 3′-UTR mutant (*MMP3* 3′UTR-MUT) were cloned into luciferase reporter plasmid psiCHECK2 and co-transfected with or without *miR-17-5p* mimics (Figure 5C). It was demonstrated that *miR-17-5p* mimics co-transfection significantly reduced the luciferase activity of the wild-type *lncRSPH9-4*, but not that of the *lncRSPH9-4* MUT (Figure 5B). Similarly, the *miR-17-5p* co-transfection also significantly decreased the luciferase activity of the wild-type *MMP3* 3′UTR, but not that of the mutant *MMP3* 3′UTR (Figure 5C). These results indicated the *lncRSPH9-4*-involved ceRNA working mechanism and supported the regulative relationship between *lncRSPH9-4* and *miR-17-5p*, as well as between *miR-17-5p* and *MMP3*.

### 2.6. LncRSPH9-4 Positively Regulated MMP3 Expression

**Figure 6:**
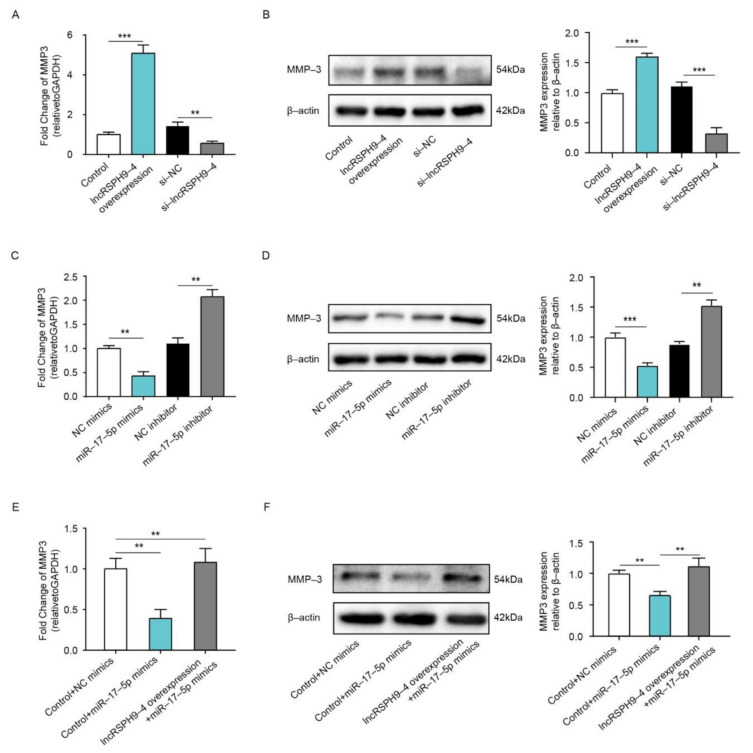
*LncRSPH9-4* overexpression increased MMP3 expression via *miR-17-5p*. (**A**,**B**) The transcription and expression levels of MMP3 in hBMECs with *lncRSPH9-4* overexpression or silencing by qPCR and Western blot analyses. *GAPDH* was used as the reference control for the qPCR, β-actin was the loading control for the blotting and densitometry was performed to analyze differences. The qPCR data were presented as mean ± SD from three independent assays. ** *p* < 0.01; *** *p* < 0.001. (**C**,**D**) The transcription and expression levels of MMP3 in hBMECs with miR-17-5p overexpression or inhibition by qPCR and Western blot analyses. *GAPDH* was used as the reference control for the qPCR, β-actin was the loading control for the blotting and densitometry was performed to analyze differences. The qPCR data were presented as mean ± SD from three independent assays. ** *p* < 0.01; *** *p* < 0.001. (**E**,**F**) The transcription and expression levels of MMP3 in hBMECs with *miR-17-5p* mimics transfection or with both *miR-17-5p* mimics and *lncRSPH9-4* co-transfection, determined by qPCR and Western blotting. *GAPDH* was used as the reference control for the qPCR, β-actin was the loading control for the blotting and densitometry was performed to analyze differences. The qPCR data were presented as mean ± SD from three independent assays. ** *p* < 0.01.

We next validated the regulative effects of *lncRSPH9-4* and *miR-17-5p* on MMP3 expression. As demonstrated, *lncRSPH9-4* overexpression significantly increased mRNA transcription and protein expression of MMP3, while, in contrast, the siRNA knocking-down of *lncRSPH9-4* significantly decreased MMP3 expression (Figure 6A,B). Meanwhile, *miR-17-5p* mimics significantly decreased the transcription, as well as expression, of MMP3. However, the *miR-17-5p* inhibitor resulted in a significant increase in the MMP3 level (Figure 6C,D). Notably, we observed that the *miR-17-5p* mimics-caused decrease of MMP3 could be entirely restored by the overexpression of *lncRSPH9-4* (Figure 6E,F), indicating that *lncRSPH9-4* positively regulated MMP3 expression via competitively sponging *miR-17-5p*.

### 2.7. LncRSPH9-4 Disrupted BBB Junction Protein through miR-17-5p/MMP3 Axis

**Figure 7:**
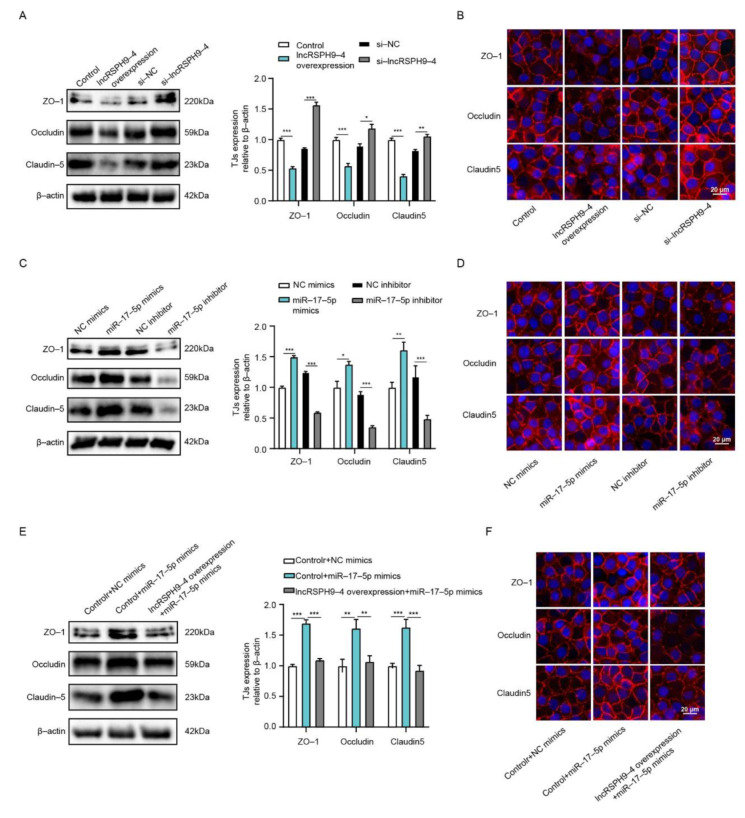
*LncRSPH9-4* overexpression decreased TJs expression via *miR-17-5p*. (**A**) The expression of TJs in hBMECs with *lncRSPH9-4* overexpression or silencing by Western blot analysis. β-actin was used as the loading control and densitometry was performed to analyze differences. * *p* < 0.05, ** *p* < 0.01 and *** *p* < 0.001. (**B**) Distribution of TJs in hBMECs with *lncRSPH9-4* overexpression or silencing by immunofluorescence analysis. (**C**) The expression of TJs in hBMECs with *miR-17-5p* overexpression or inhibition by Western blot analysis. β-actin was used as the loading control and densitometry was performed to analyze differences. * *p* < 0.05, ** *p* < 0.01 and *** *p* < 0.001. (**D**) Distribution of TJs in hBMECs with *miR-17-5p* overexpression or inhibition by immunofluorescence analysis. (**E**) The expression of TJs in *miR-17-5p*-overexpressed hBMECs co-transfected with or without *lncRSPH9-4* by Western blot analysis. β-actin was used as the loading control and densitometry was performed to analyze differences. ** *p* < 0.01 and *** *p* < 0.001. (**F**) Distribution of TJs in *miR-17-5p*-overexpressed hBMECs co-transfected with or without *lncRSPH9-4* by immunofluorescence analysis.

Previous studies have reported that MMP3 contributed to the disruption of TJs [25,26]. We subsequently determined whether *lncRSPH9-4* participated in decreasing the TJs in hBMECs through *miR-17-5p*/MMP3 axis. By Western blotting analyses, we found that *lncRSPH9-4* negatively regulated the expression of TJs, by the demonstrations that *lncRSPH9-4* overexpression caused a significant decrease of ZO-1, Occludin and Claudin-5, while silencing *lncRSPH9-4* significantly increased their protein levels (Figure 7A). In addition, immunofluorescence assays were performed to further examine the effect of *lncRSPH9-4* on the BBB integrity. As shown in Figure 7B, the levels of TJs between adjacent endothelial cells were significantly decreased upon overexpression of *lncRSPH9-4* and silencing *lncRSPH9-4* well maintained the morphology of TJs. Correspondingly, the effects of *miR-17-5p* on the expression of TJs were also determined and the results showed an obvious positive-regulation on the expression of TJs, by the observations that *miR-17-5p* mimics increased the expression of ZO-1, Occludin and Claudin-5, while *miR-17-5p* inhibitors significantly decreased their expression (Figure 7C). Likewise, by immunofluorescence analysis, we demonstrated that TJs between adjacent endothelial cells could be well maintained when overexpressing *miR-17-5p*, whereas transfection with the *miR-17-5p* inhibitor resulted in a significant breakdown of TJs (Figure 7D). More importantly, the up-regulative effects of *miR-17-5p* mimics on TJs were primarily reversed by the overexpression of *lncRSPH9-4* (Figure 7E), which was consistent with the regulation mode of *lncRSPH9-4/miR-17-5p* on MMP3. The immunofluorescence assays also showed that the up-regulative effects of *miR-17-5p* mimics on hBMECs monolayer integrity were well blocked when *lncRSPH9-4* was overexpressed (Figure 7F). Taken together, these findings suggested that *lncRSPH9-4* aggravated the TJs disruption in hBMECs by sponging *miR-17-5p* to regulate MMP3 expression, which eventually broke the barrier integrity and increased the permeability of hBMECs.

## 3. Discussion

Due to their extensive involvement in biological processes, lncRNAs have attracted immense research interest in recent years. LncRNAs have been increasingly involved in the regulation of multiple central nervous system disorders, such as ischemic stroke, multiple sclerosis and Huntington’s disease [16,27,28]. However, in CNS infectious diseases, the regulatory function of lncRNAs was largely unclear. In this study, we identified and characterized an up-regulated lncRNA, *lncRSPH9-4*, in hBMECs challenged with meningitic *E. coli*. We demonstrated that *lncRSPH9-4* helped the infection-caused disruption of the BBB integrity via sponging *miR-17-5p*, thus promoted the expression of MMP3 and eventually increased the degradation of TJs.

Lots of lncRNAs were involved in the development of neurodegenerative diseases. A well-studied lncRNA, *MALAT1*, was reported to contribute to protecting the BBB after stroke [29]. Other lncRNAs, such as *BC200* and *Sox2OT*, were found to be associated with Alzheimer’s disease or Parkinson’s disease [30,31]. Accumulating pieces of evidence have also supported the essential roles of lncRNAs in virus infection. For example, a lncRNA called lncRNA-ACOD1 was found to facilitate *Vesicular vtomatitis virus* replication in macrophages through enhancing GOT2 enzymatic activity [12]. LncRNAs exerted their functions from many aspects. In recent years, the concept of ceRNA as a new regulatory mechanism has been raised and widely accepted in diverse physiological and pathological processes, which means that lncRNAs could act as a ceRNA to competitively sponge miRNA, thus resulting in a decreased mRNA degradation [32]. Numerous studies have increasingly reported that lncRNAs function as sponges to interact with miRNAs at the post-transcriptional level [33,34]. For example, a lncRNA named *linc-EPHA6-1* can sponge *miR-4485-5p* to regulate NKP46 expression and enhance NK cells cytotoxicity against *Zika virus*-infected cells [35]. However, there are few studies about the lncRNAs function in bacterial infectious diseases. We previously found one lncRNA, *lncRSPH9-4*, that was significantly increased in meningitic *E. coli*-infected hBMECs. Here, we further analyzed the transcriptomic profiles in hBMECs in response to the overexpression of *lncRSPH9-4* via RNA-sequencing. A total of 639 mRNAs and 299 miRNAs with significant alteration upon lncRSPH9-4 overexpression have been identified and the ceRNA regulatory network was built based on the sequencing data. One miRNA, *miR-17-5p*, showed the possibility of being sponged by *lncRSPH9-4* and influenced the expression of MMP3, an important protein associated with intercellular integrity and permeability.

It is worth wondering about the relationship between *miR-17-5p* and the BBB permeability. The BBB is ultrastructurally assembled by a monolayer of BMECs, which are tightly attached via TJs and adherens junctions (AJs) [6,36]. Many miRNAs have been shown to influence the BBB permeability. Some pathogens, such as *Coxsackievirus A16*, can increase the expression of *miRNA-1303* and trigger the changes in the BBB permeability [37]. Other miRNAs, such as *miRNA-29b* and *miRNA-15*, were reported to control the BBB integrity by targeting MMP9 [38,39]. Exosomes-derived *miRNA-132* regulated the expression of vascular endothelial cadherin (VE-cadherin) by directly targeting eukaryotic elongation factor 2 kinase (eef2k) and increased the permeability of the BBB [40]. It has been reported that *miR-17-5p* plays a vital role in endothelial cells by binding to *VEGF-A* 3′UTR and inhibiting *VEGF-A* expression in HUVECs [41]. Here, in our study, we used an immortalized hBMECs cell line, established by Kwang Sik Kim in Johns Hopkins University School of Medicine, as our in vitro BBB model. This immortalized hBMECs line was positive for Factor VIII-Rag, could intake DiI-AcLDL and showed a positive reaction for GGTP, which indicated that they exhibited brain endothelial characteristics [42]. It also expressed VCAM-1, ICAM-1 and TJs and maintained their barrier properties [43]. The hBMECs monolayer model provided by Kim has been largely applied in BBB functional research. For example, molecules such as CC chemokine ligand 2 (CCL2) and snail family transcriptional repressor 1 (Snail1) have been reported to contribute to blood–brain barrier disruption by the application of the hBMEC monolayer [44,45]. In our study, we found that *miR-17-5p* expression was significantly downregulated in hBMECs when overexpressing *lncRSPH9-4*. Consistently, *miR-17-5p* was also decreased in hBMECs in response to meningitic *E. coli* infection, as observed in our previous work [46]. Here, we showed that *miR-17-5p* was one of the competitive-sponging targets of *lncRSPH9-4* and *miR-17-5p* could bind to 3′UTR of MMP3 to suppress the expression of MMP3. MMP3 is a member of the MMP family, which is widely involved in the breakdown of extracellular matrix proteins during tissue remodeling, such as embryonic development and reproduction, as well as in disease processes, such as arthritis and tumor metastasis [47]. MMP3 can degrade collagen, elastin, fibronectin, laminin, as well as tight junctions, so as to increase vascular permeability and disrupt the BBB [25,41,48,49]. In hBMECs, our results suggested that *lncRSPH9-4* was able to regulate MMP3 expression by acting as a competitive sponge of *miR-17-5p*. Since previous studies have already evidenced that MMP3 can directly degrade the TJs of vascular endothelial cells [25,41], we therefore next investigated the potential effects on MMP3 regulated by *lncRSPH9-4* and *miR-17-5p* and results showed that *lncRSPH9-4* could negatively regulate the TJs, while *miR-17-5p* positively regulated the TJs and, importantly, this regulatory axis was probably mediated by the change of MMP3 expression during this process.

**Figure 8:**
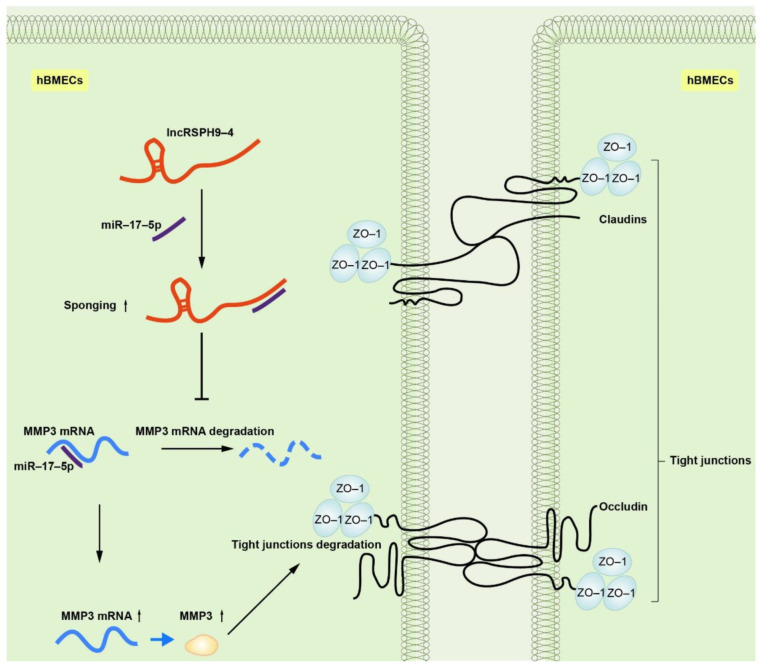
Schematic of the working mechanism of *lncRSPH9-4* in the regulation of the endothelial barrier disruption. Meningitic *E. coli* infection of hBMECs induced the up-regulation of *lncRSPH9-4*, which could sponge *miR-17-5p* and thus increase the expression of MMP3 in hBMECs and finally lead to the tight junction degradation.

Taken together, our findings provided substantial evidence, for the first time clarifying the regulatory function of lncRNA during meningitis-causing bacterial infection. We demonstrated that meningitic *E. coli*-induced *lncRSPH9-4* could function as a ceRNA to compete with *miR-17-5p*, which led to the increased expression of MMP3 and therefore the degradation of the TJs barrier (Figure 8). However, the mechanisms of BBB disruption in the development of bacterial meningitis are quite complex and the contribution of *lncRSPH9-4* is just the tip of the iceberg. At the same time, we investigated the expression specificity of *lncRSPH9-4* in the other endothelial cells (peripheric endothelial cells, such as HUVECs), as well as other barrier-forming (not endothelium) cells, such as astrocytes, in our previous work. As demonstrated below, *lncRSPH9-4* was also significantly induced by the challenge of meningitic *E. coli*, indicating that *lncRSPH9-4* probably exhibits a similar working mechanism in the astrocytes cell line U251. We also noticed the expression/transcription of *lncRSPH9-4* in the peripheric vascular endothelial cells HUVECs; however, it was not significantly induced by the infection, suggesting that *lncRSPH9-4* might not work in this type of endothelial cells [20]. At last, further efforts are required to explore the generation of *lncRSPH9-4* and whether there are some other targets as well as regulative mechanisms of *lncRSPH9-4* in meningitic bacterial penetration of the BBB.

## 4. Materials and Methods

### 4.1. Cell Line and Cell Culture

The immortalized hBMECs cell line was kindly provided by Kwang Sik Kim, at Johns Hopkins University School of Medicine. The immortalized hBMECs were obtained by transfecting the pBR322 construct containing simian virus large T protein (SV-large T). This hBMECs cell line was authenticated by the Genetic Testing Biotechnology Corporation (Suzhou, China), using short tandem repeat (STR) markers. It was routinely cultured in RPMI1640 supplemented with 10% fetal bovine serum (FBS), 2mML-glutamine, 1 mM sodium pyruvate, essential amino acids, nonessential amino acids, vitamins and penicillin and streptomycin (100 U/mL), in a 37 °C-incubator under 5% CO2 until monolayer confluence.

### 4.2. Meningitic E. coli Infection of hBMECs

The meningitic *E. coli* strain used in this study, PCN033 [Genbank: CP006632.1], was isolated from swine cerebrospinal fluid in China. The strain was routinely grown aerobically at 37 °C in Luria-Bertani (LB) medium overnight. PCN033-induced infections of hBMECs were performed as follows. An overnight *E. coli* culture was resuspended and diluted in serum-free medium and then it was added to a starved confluent hBMEC monolayer grown in 10 cm dishes at an MOI of 10 (approximately 10^8^ colony-forming units per dish) to allow invasion at 37 °C for 3 h.

### 4.3. Transfection

The hBMECs were cultured until 50%–60% confluence in a 6-well plate and transfected with pcDNA3.1 (+)-*lncRSPH9-4*, which was purchased from Genscript (Genscript, Nanjing, China) using the jetPRIME transfection reagent (Polyplus transfection, Illkirch, France), according to the manufacturers’ instructions. Briefly, 200 μL jetPRIME buffer was added into the pcDNA3.1 (+)-*lncRSPH9-4* plasmid before the addition of 4 μL of jetPRIME. The suspension was shortly mixed by vortexing, incubated at room temperature for 10 min and then added dropwise to the cells and incubated at 37 °C with 5% CO_2_ for 48 h.

### 4.4. Electrical Cell-Substrate Impedance Sensing 

To evaluate the real-time alteration of the monolayer cell resistance, the ECIS system (Applied BioPhysics, Troy, MI, USA) was applied to compare the impedance values in hBMECs with or without *lncRSPH9-4* overexpression seeded on the collagen-coated, gold-plate electrodes in 96-well chanmer slides (96W1E+), as previously described [50]. Two ECIS parameters, R (Ω), representing the electrical cell–cell contacts, and Rb (Ωcm^2^), representing the paracellular barrier, were extracted from the continuously recorded impedance spectra to reflect the real-time changes of the monolayer barrier function.

### 4.5. RNA Isolation and Quantitative Real-Time PCR Analysis

Total cellular RNA was extracted using the TRIzol reagent (Invitrogen, Carlsbad, CA, USA) in hBMECs, after being washed three times with chilled PBS buffer. For miRNAs, cDNA synthesis was carried out using miRcute plus miRNA First-Strand cDNA kit (Tiangen, Beijing, China), according to the manufacturer’s instructions. The qPCR was performed using the miRcute plus miRNA qPCR detection kit (Tiangen, Beijing, China). Primers for the miRNA qPCR are listed in Supplementary Table S1. For mRNAs, aliquots (500 ng) of the total RNAs were reverse-transcribed into cDNA using the HiScript II Q RT SuperMix (Vazyme, Nanjing, China). Real-time PCR was performed with a qTOWER3/G quantitative real-time PCR thermal cycler (Analytikjena, Jena, Germany) with the AceQ qPCR SYBR Green Master Mix (Vazyme, Nanjing, China), according to the manufacturer’s instructions. The amplification conditions were 50 °C for 2 min, 95 °C for 10 min, followed by 40 cycles of 95 °C for 15 s and 60 °C for 1 min. The products were then subjected to a melting curve stage comprising denaturation at 95 °C for 15 s, annealing at 60 °C for 1 min and slow dissociation by ramping from 60 °C to 95 °C at 0.1 °C/s to ensure the specificity of the primers for their target sequences. Expression of the target genes was normalized against *GAPDH*. Each assay was performed with three replicates.

### 4.6. mRNA Library Preparation and Sequencing

Six RNA sequencing libraries were constructed and 150-bp paired-end sequencing was applied with the Illumina HiSeq platform. RNA sequencing libraries were prepared from 2 μg of total RNAs using the TruSeq kit (Illumina, San Diego, CA, USA) with the following modification. Instead of purifying poly-A RNA using poly-dT primer beads, we removed ribosomal RNA using the Ribo-Zero rRNA Removal kit (Illumina, San Diego, CA, USA). All other steps were performed according to the manufacturer’s protocols. RNA-seq libraries were analyzed for quality control and the average size of inserts was approximately 200–300 bp. The sequencing library was then sequenced on a Hiseq platform (Illumina, San Diego, CA, USA) by Shanghai Personal Biotechnology Cp. Ltd. (Personal Biotechnology, Shanghai, China).

### 4.7. Small RNA Library Preparation and Sequencing

Small RNA libraries were constructed using the NEBNext Multiplex Small RNA Library Prep Set for Illumina (New England Biolabs, Inc.). The total RNA from six samples were mixed with 3′ linker sequences, RT Primers and the 5′ adaptor sequences. The resulting RNA samples were reverse-transcribed by following these steps: degeneration at 94 °C for 15 s, annealing at 62 °C for 30 s and extension at 70 °C for 15 s. Small RNA libraries were analyzed for quality control and the average size of inserts was about 140–150 bp. The library was then subjected to the sequencing on a Hiseq platform (Illumina, San Diego, CA, USA) by Shanghai Personal Biotechnology Cp. Ltd. (Personal Biotechnology, Shanghai, China).

### 4.8. Construction of the lncRNA-miRNA-mRNA ceRNA Network

The lncRNA–miRNA–mRNA ceRNA network was based on the theory that lncRNAs can directly interact by invoking miRNA sponges to regulate mRNA activity. To investigate the potential interactions between lncRNAs and mRNAs, we constructed the ceRNA co-regulated network using differentially expressed lncRNAs, differentially expressed miRNA and differentially expressed mRNA data. The co-regulated network was constructed using Cytoscape software version 3.7.0 (http://www.cytoscape.org) to illustrate the ceRNA network.

### 4.9. Dual-Luciferase Reporter Assays

Until 50–70% of confluence, HEK293T cells were co-transfected with 100 ng psiCheck-2 *lncRSPH9-4* or *MMP3* 3′ UTR luciferase reporter plasmid along with *miR-17-5p* mimics or controls (final concentration, 50 nM). HEK293T cells were co-transfected with 100 ng psiCheck-2 mutant *lncRSPH9-4* or mutant MMP3 3′ UTR luciferase reporter plasmid lacking the *miR-17-5p* binding site along with *miR-17-5p* mimics or controls (final concentration, 50 nM). At 24 h post-transfection, the reporter luciferase activities were measured using the Dual-Luciferase reporter assay system (Promega, Madison, WI, USA), following the manufacturers’ instruction.

### 4.10. FISH

The commercial FISH Kit, purchased from GenePharma (Shanghai, China), was used for the FISH assays, following the manufacturer’s procedures. Briefly, hBMECs were fixed in 4% paraformaldehyde for 30 min at room temperature and washed twice with PBS. Cells were incubated in proteinase K solution for 15 min at 37 °C, washed twice with PBS for 5 min and then mixed with 1% fixation solution for 10 min at room temperature. Fixed samples were incubated with 70% ethanol for 5 min, 85% ethanol for 5 min and 100% ethanol for 5 min at −20 °C, before drying. Samples were next incubated with a pre-hybridization solution for 30 min at 37 °C, followed by incubating in the hybridization buffer with specific probes for *lncRSPH9-4* (5′-Cy3-AGGAAAGAGGCTCAGGCCCA-3′) for 18 h at 37 °C. After strict wash in SSC buffer, 1mg/mL of 4′,6-diamidino-2-phenylindole (DAPI, Beyotime Biotechnology, Shanghai, China) was used for nucleus staining. Fluorescent images were acquired with an OlympusFV10 laser scanning confocal microscope (Olympus, Tokyo, Japan).

### 4.11. Western Blotting

The hBMECs transfected with pcDNA3.1-*lncRSPH9-4*, si-*lncRSPH9-4*, or *miR-17-5p* mimics, or *miR-17-5p* inhibitors were lysed in RIPA buffer with a protease inhibitor cocktail (Sigma-Aldrich, St. Louis, MO, USA), sonicated and centrifuged at 10,000 g for 10 min at 4 °C. The insoluble debris was removed and protein concentration in the supernatant was measured using the BCA protein assay kit (NCM Biotech, Suzhou, China). The protein samples were then separated on 12% sodium dodecyl sulfate-polyacrylamide gel electrophoresis (SDS-PAGE) and transferred to polyvinylidene difluoride (PVDF) membranes. The blots were blocked in 5% BSA in Tris-buffered saline with Tween 20 (TBST) for 2 h at room temperature and then incubated overnight with primary antibodies against ZO-1, Occludin, Claudin-5, MMP3 (Abcam, Cambridge, MA, USA), MMP1, β-actin (Proteintech, Chicago, IL, USA), or MMP10 (ABclonal, Wuhan, China). The blots were subsequently washed and incubated with horseradish peroxidase-conjugated anti-rabbit or anti-mouse IgG (Biodragon, Beijing, China) at 37 °C for 1 h. The blots were visualized with the Super electrochemiluminescence (ECL) Prime kit (US Everbright, Suzhou, China) and were densitometrically analyzed using the Image Lab software (Bio-Rad, Hercules, CA, USA).

### 4.12. Immunofluorescence Assay

The hBMECs grown in 12-well dishes were fixed with 4% paraformaldehyde for 15 min, followed by three washes with PBS. Cells were incubated with the primary rabbit ZO-1, Occludin, or Claudin-5 antibody (Abcam, Cambridge, MA, USA) overnight at 4 °C and then incubated with Cy3 goat anti-rabbit antibody (Proteintech, Chicago, IL, USA) for 1 h. Cells were counterstained with DAPI to visualize the nucleus morphology and mounted and photographed using the BX41 fluorescence microscopy (Olympus, Tokyo, Japan).

### 4.13. Statistical Analysis

Data were expressed as mean ± SD from three independent experiments and the significance of differences between groups was evaluated by one-way analysis of variance. * *p* < 0.05 was considered significant, ** *p* < 0.01 and *** *p* < 0.001 were all considered extremely significant. Graphs were plotted and analyzed using GraphPad Prism version 6.0 (GraphPad Software, La Jolla, CA, USA).

## 5. Conclusions

This study provided novel insights into the molecular mechanism of *lncRSPH9-4* in meningitic *E. coli* invasion of the BBB. We demonstrated that meningitic *E. coli* infection of hBMECs induced the significant upregulation of *lncRSPH9-4*, which facilitated the barrier disruption of the endothelial cells, probably through *lncRSPH9-4*/*miR-17-5p*/MMP3 axis. This finding provides a new idea for understanding the function of lncRNAs in bacterial infection, especially in bacterial penetration of the CNS. Blocking these lncRNAs, such as *lncRSPH9-4*, may also represent a new strategy to better prevent and improve the CNS dysfunctions.

## Data Availability

The data presented in this study are available on request from the corresponding author.

## Supplementary Materials

The following are available online at https://www.mdpi.com/article/10.3390/ijms22126343/s1, Figure S1: The construction and transfection efficiency of the *lncRSPH9-4* overexpression plasmid in hBMECs, Figure S2: The KEGG and GO enrichment analyses of the differential mRNAs in hBMECs with the *lncRSPH9-4* overexpression, Figure S3: The KEGG and GO enrichment analyses presenting the differential mRNA targets of the differential miRNAs in hBMECs with *lncRSPH9-4* overexpression. Table S1 Primers used for miRNA qPCR.
