# Supplementary material for: LncRSPH9-4 Facilitates Meningitic Escherichia coli-Caused Blood–Brain Barrier Disruption via miR-17-5p/MMP3 Axis
Source: Int J Mol Sci. 2021 Jun 14;22(12):6343. doi: 10.3390/ijms22126343 (PMC8231991; doi:10.3390/ijms22126343)
Supplement: Supplementary file 1 [file ijms-22-06343-s001.zip › Supplementary Figures .pdf]

Supplement Figures

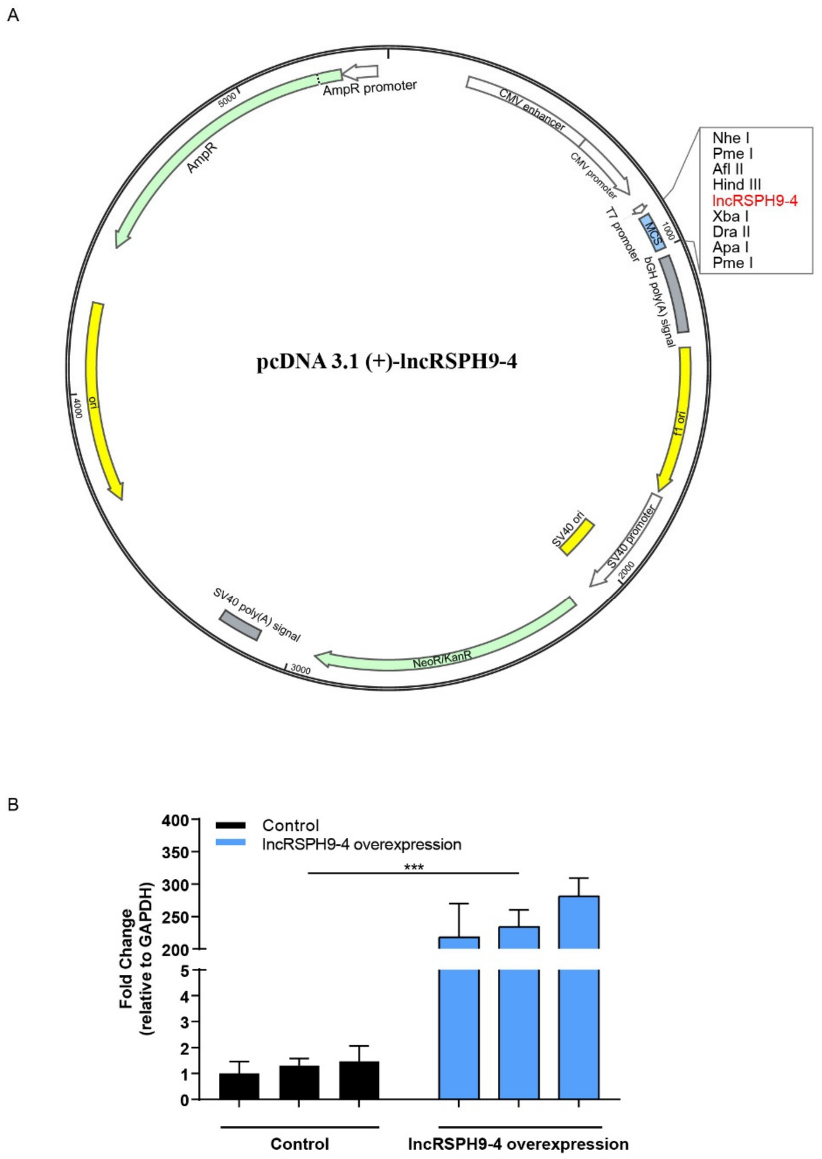

Figure S1. The construction and transfection efficiency of the lncRSPH9-4 overexpression plasmid in hBMECs. (A) Construction schematic of the lncRSPH9-4 in pcDNA3.1(+). (B) The qPCR analysis of the transfection efficiency of the lncRSPH9-4 overexpression plasmid in hBMECs. The X-axis represents three replicates of the lncRSPH9-4 overexpressed hBMECs and three control cells.

A

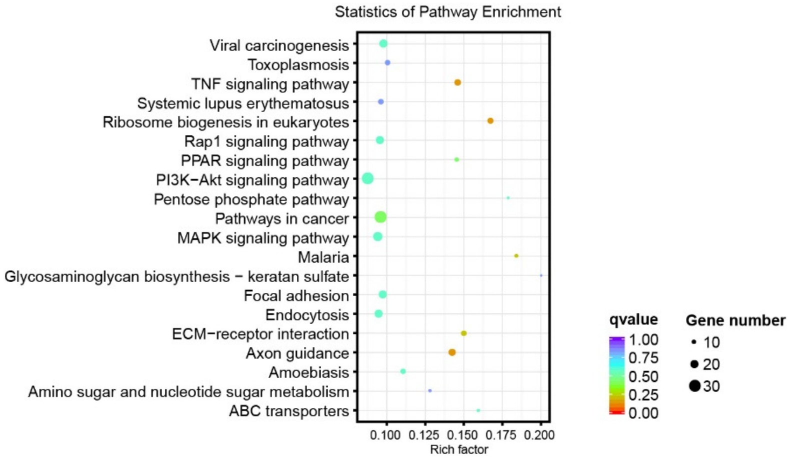

B

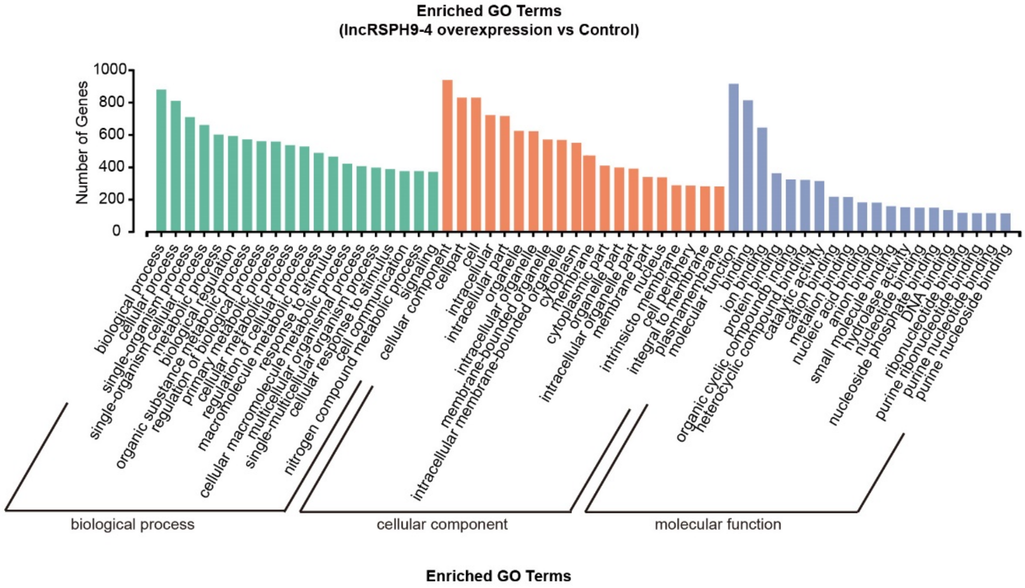

Figure S2. The KEGG and GO enrichment analyses of the differential mRNAs in hBMECs with the lncRSPH9-4 overexpression. (A) Kyoto Encyclopedia of Genes and Genomes (KEGG) pathway enrichment analysis of the differentially expressed mRNAs. The X-axis represents the rich factor, and the Y-axis represents the enriched pathways. The dots color indicates the significance of the rich factor, and their sizes are proportional to the number of genes. (B) Gene ontology (GO) enrichment analysis of the differentially expressed mRNAs.

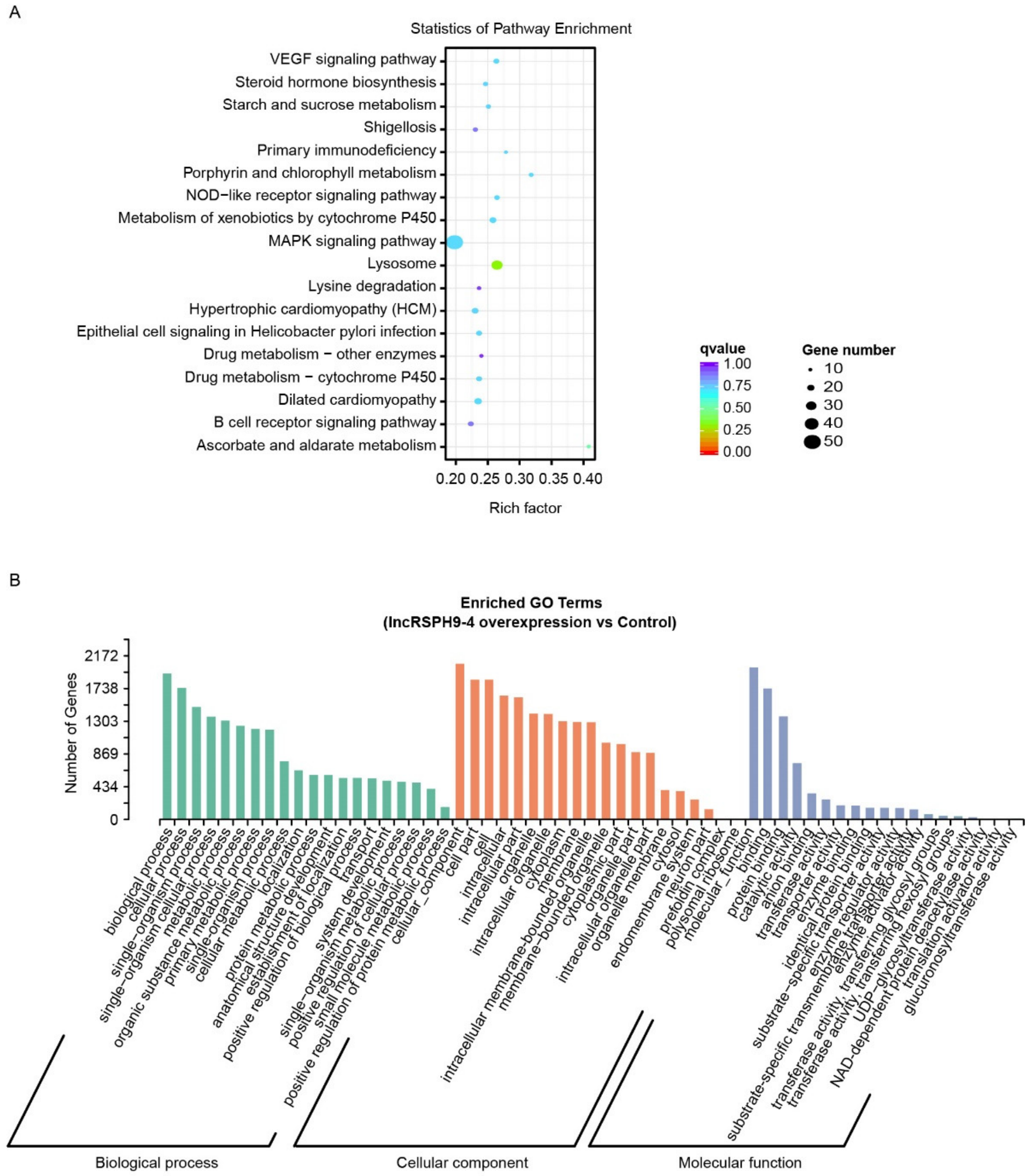

Figure S3. The KEGG and GO enrichment analyses presenting the differential mRNA targets of the differential miRNAs in hBMECs with lncRSPH9-4 overexpression. (A) KEGG pathway enrichment of the differential mRNA targets of those differential miRNAs. (B) GO enrichment analysis of the differentially expressed mRNA targets of those differential miRNAs.
