# Supplementary material for: LncRSPH9-4 Facilitates Meningitic Escherichia coli-Caused Blood–Brain Barrier Disruption via miR-17-5p/MMP3 Axis
Source: Int J Mol Sci. 2021 Jun 14;22(12):6343. doi: 10.3390/ijms22126343 (PMC8231991; doi:10.3390/ijms22126343)
Supplement: Supplementary file 1 [file ijms-22-06343-s001.zip › Suuplementary Table S1.pdf]

Supplementary Table S1 Primers used for miRNA qPCR

Primers	Nucleotide sequence (5'-3')	Amplified fragments
P1	TTCACAGTGGCTAAGTTCTGC	has-miR-27b-3p
P2	CAAAGTGCTGTTCGTGCAGGTAG	has-miR-93-5p
P3	TGTAAACATCCTACACTCAGCT	hsa-miR-30b-5p
P4	AGGGACTTTTGGGGGCAGATGTG	hsa-miR-365a-5p
P5	TGTTGGGATTCAGCAGGACCAT	hsa-miR-4425
P6	CAGTGCCTCGGCAGTGCAGCCC	hsa-miR-33b-3p
P7	AGCCCCTGCCCACCGCACACTG	hsa-miR-210-5p
P8	CAAAGTGCTTACAGTGCAGGTAG	hsa-miR-17-5p
P9	TCTTTGGTTATCTAGCTGTATGA	hsa-miR-9-5p
P10	CAATGTTTCCACAGTGCATCAC	hsa-miR-33a-3p
P11	TTGATCTCGGAAGCTAAGC	hsa-miR-7641
P12	CTCTCTACTGACTTGCAACATA	hsa-miR-4684-5p
P13	AGGTTGGGATCGGTTGCAATGCT	hsa-miR-92a-1-5p
P14	AGTTCTTCAGTGGCAAGCTTTA	hsa-miR-22-5p
P15	AGCCAGGCTCTGAAGGGAAAGT	hsa-miR-4755-3p
P16	TTTCCCTTCAGAGCCTGGCTTT	hsa-miR-4755-5p
P17	TGAGAACTGAATTCCATGGGTT	hsa-miR-146a-5p
P18	CATCTTACTGGGCAGCATTGGA	hsa-miR-200b-5p
P19	GCTGGTTTCATATGGTGGTTTAGA	hsa-miR-29b-1-5p
P20	AAAAGCTGGGTTGAGAGGGT	hsa-miR-320c
