# Supplementary figures and images for: LncRSPH9-4 Facilitates Meningitic Escherichia coli-Caused Blood–Brain Barrier Disruption via miR-17-5p/MMP3 Axis
Source: Int J Mol Sci. 2021 Jun 14;22(12):6343. doi: 10.3390/ijms22126343 (PMC8231991; doi:10.3390/ijms22126343)

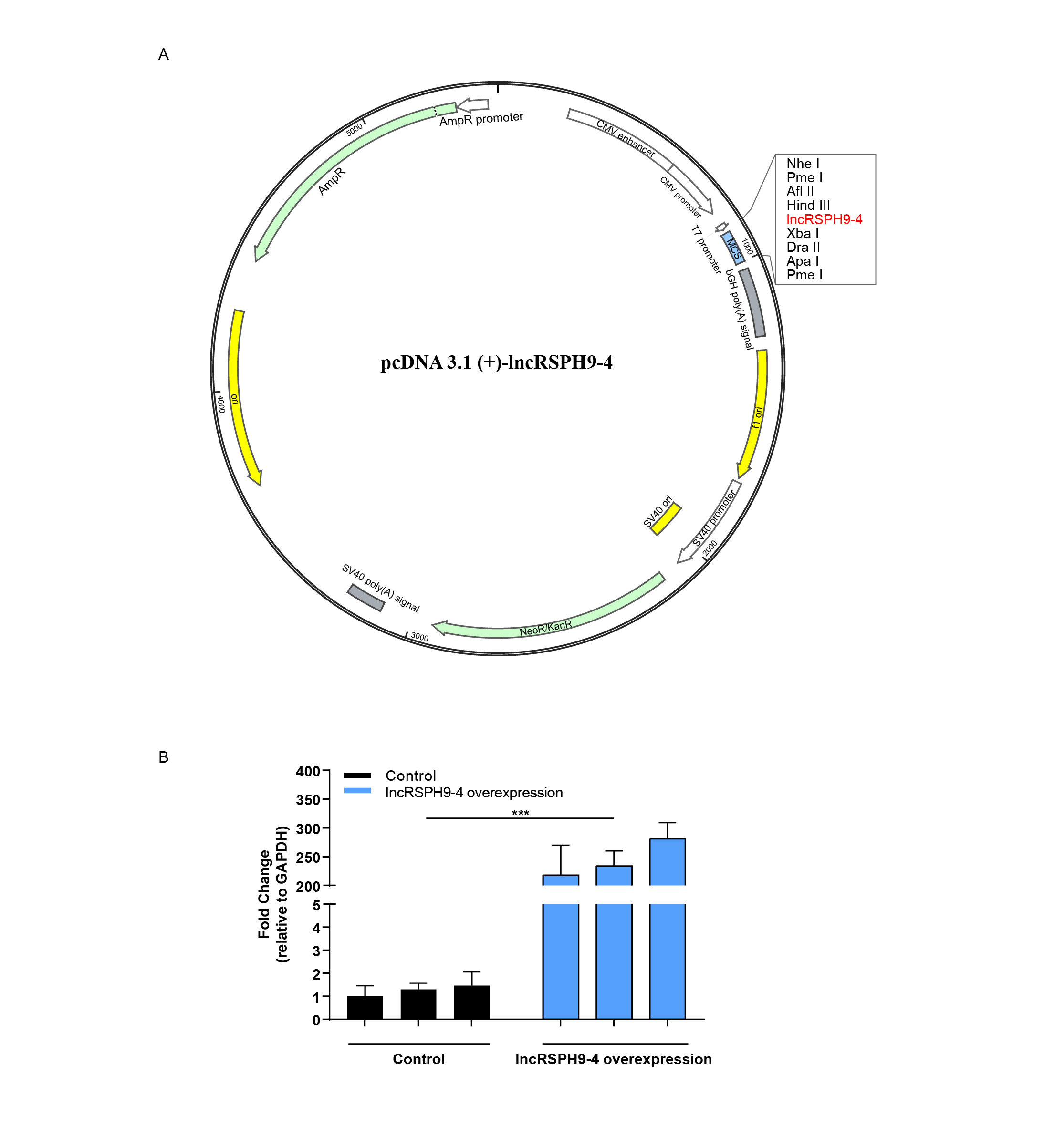

Supplement: Supplementary file 1 [file ijms-22-06343-s001.zip › FIGS1 MDPI.jpg]

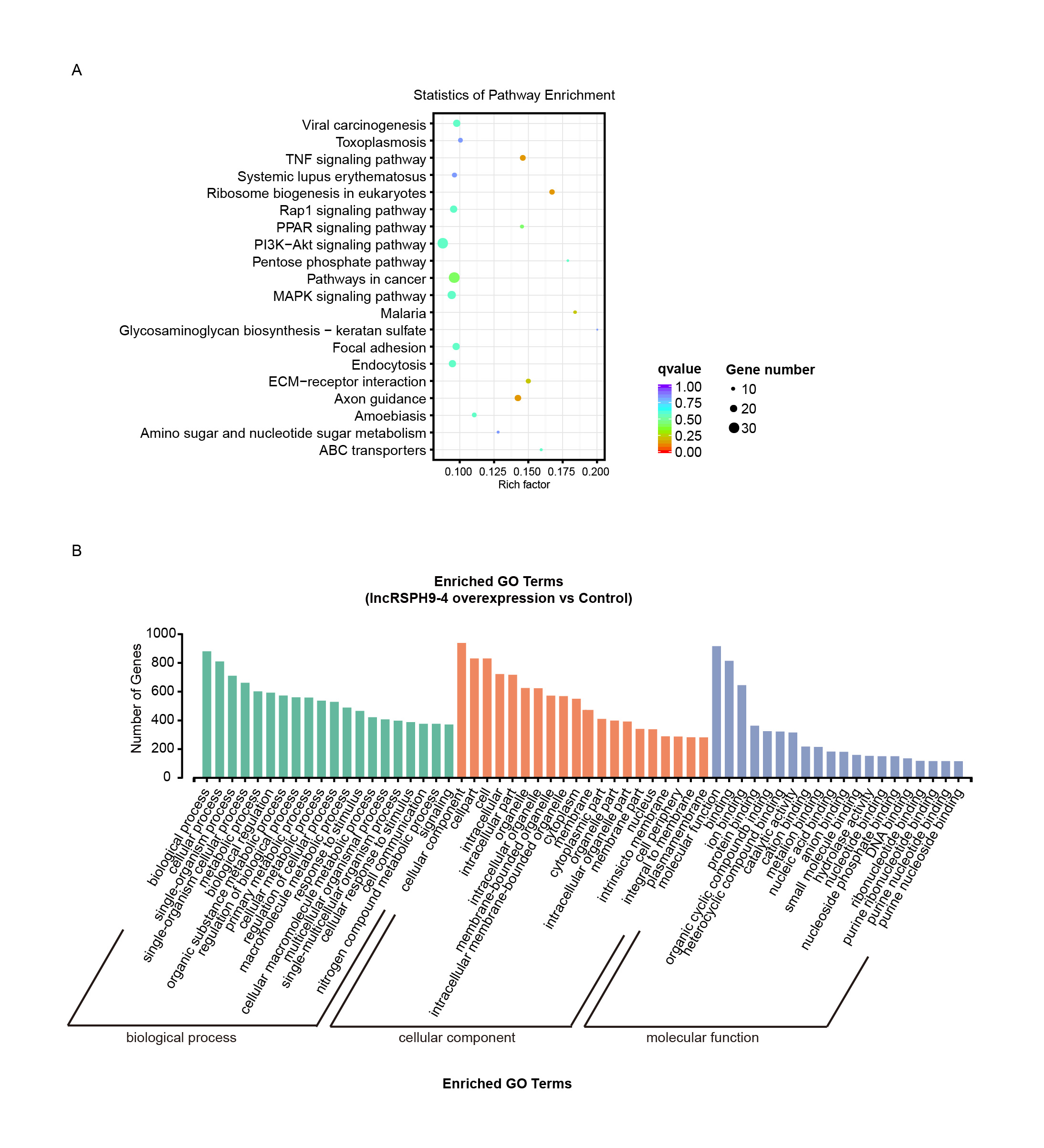

Supplement: Supplementary file 1 [file ijms-22-06343-s001.zip › FIGS2 MDPI.jpg]

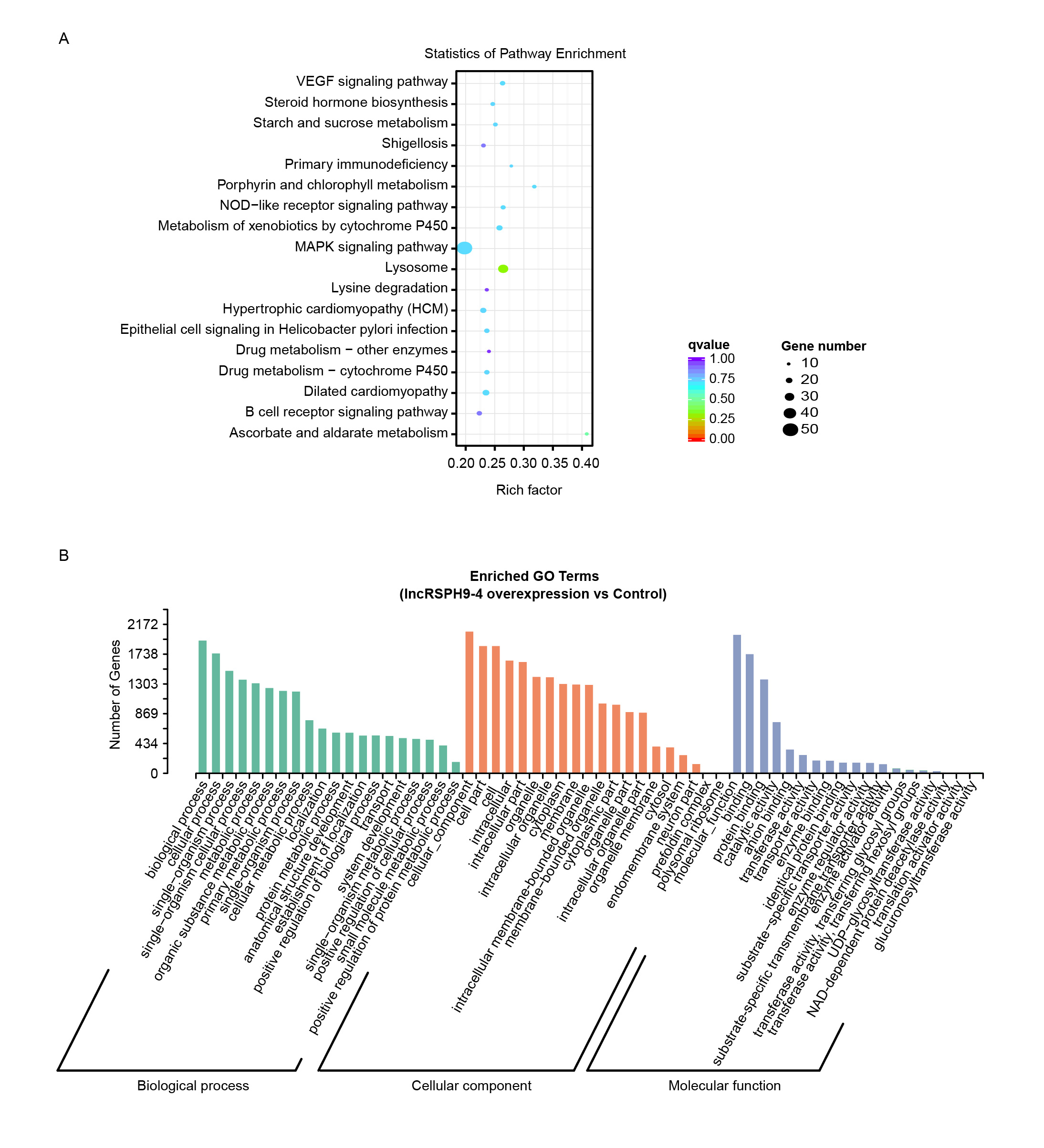

Supplement: Supplementary file 1 [file ijms-22-06343-s001.zip › FIGS3 MDPI.jpg]

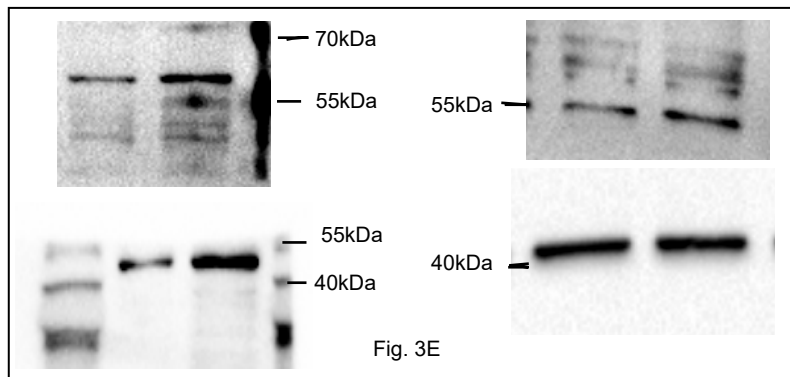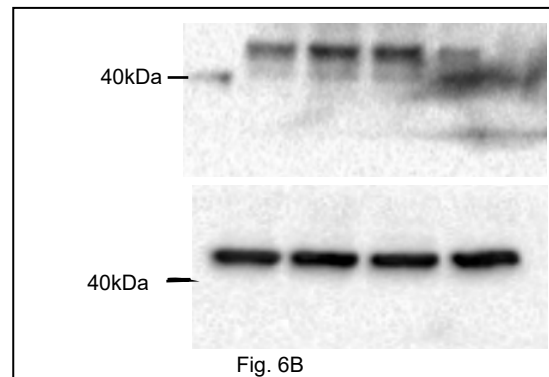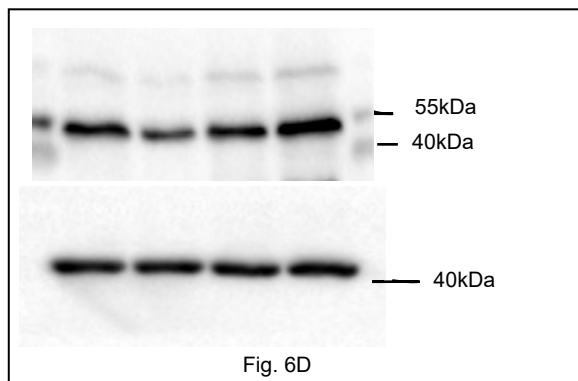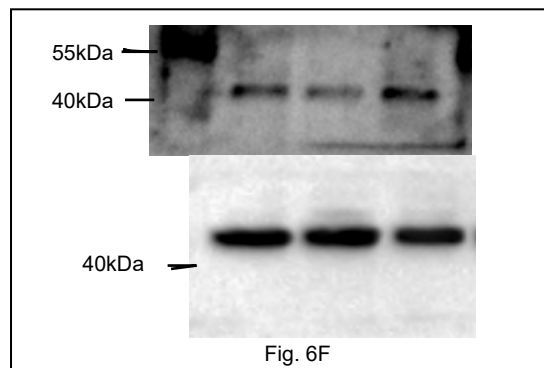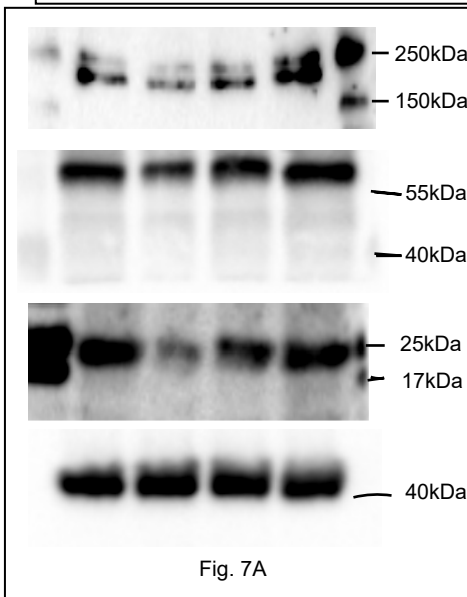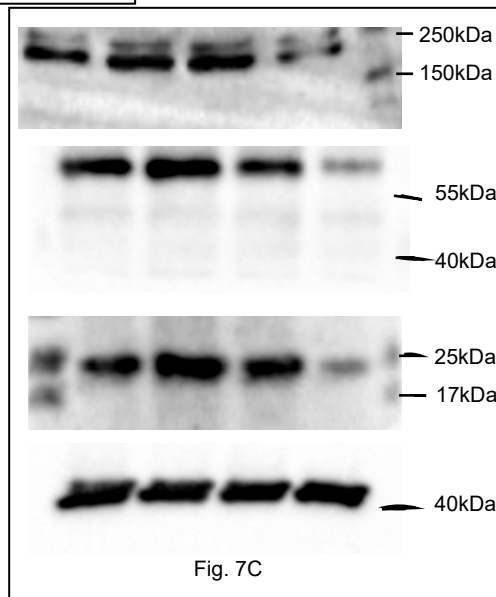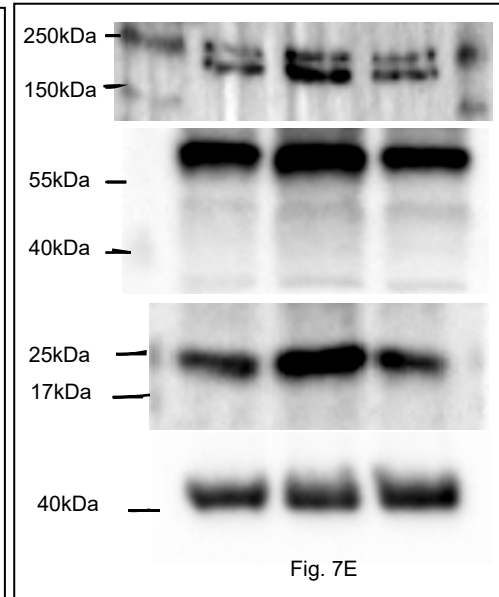

Supplement: Supplementary file 1 [file ijms-22-06343-s001.zip › Original Images.pdf]
